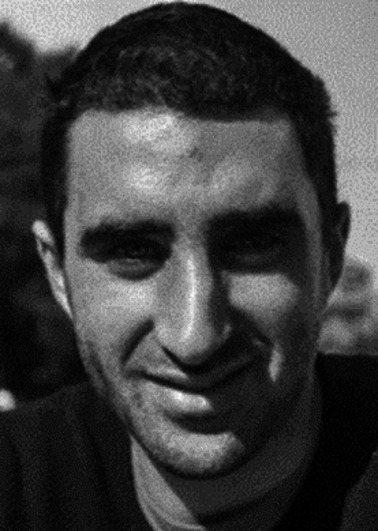# Advances in Catalytic Routes for the Homogeneous Green Conversion of the Bio‐Based Platform 5‐Hydroxymethylfurfural

**DOI:** 10.1002/cssc.202200228

**Published:** 2022-05-12

**Authors:** Alessandro Messori, Andrea Fasolini, Rita Mazzoni

**Affiliations:** ^1^ Department of Industrial Chemistry “Toso Montanari” University of Bologna Viale Risorgimento, 4 40136 Bologna Italy; ^2^ Center for Chemical Catalysis – C3 University of Bologna Viale Risorgimento, 4 40136 Bologna Italy

**Keywords:** 5-hydroxymethylfurfural, biomass valorization, homogeneous catalysis, ligands, sustainable chemistry

## Abstract

5‐Hydroxymethylfufural (HMF) is an intriguing platform molecule that can be obtained from biomasses and that can lead to the production of a wide range of products, intermediates, or monomers. The presence of different moieties in HMF (hydroxy, aldehyde, furan ring) allows to carry out different transformations such as selective oxidations and hydrogenations, reductive aminations, etherifications, decarbonylations, and acetalizations. This is a great chance in a biorefinery perspective but requires the development of active and highly selective catalysts. In this view, homogeneous catalysis can lead to efficient conversion of HMF at mild reaction conditions. This Review discussed the recent achievements in homogeneous catalysts development and application to HMF transformations. The effects of metal nature, ligands, solvents, and reaction conditions were reported and critically reviewed. Current issues and future chances have been presented to drive future studies toward more efficient and scalable processes.

## Graphical abstract

**Homogeneous catalysis**: This Review describes advances in the homogeneously catalyzed conversion of 5‐hydroxymethylfurfural (HMF) to several other bio‐based building blocks. State of the art in oxidation, reduction, reductive amination, decarbonylation, and Friedel‐Craft arylation by metal salts with co‐catalysts or molecular complexes as catalytic systems is critically analyzed to draw a perspective from both sustainability and possible development to real application point of view.

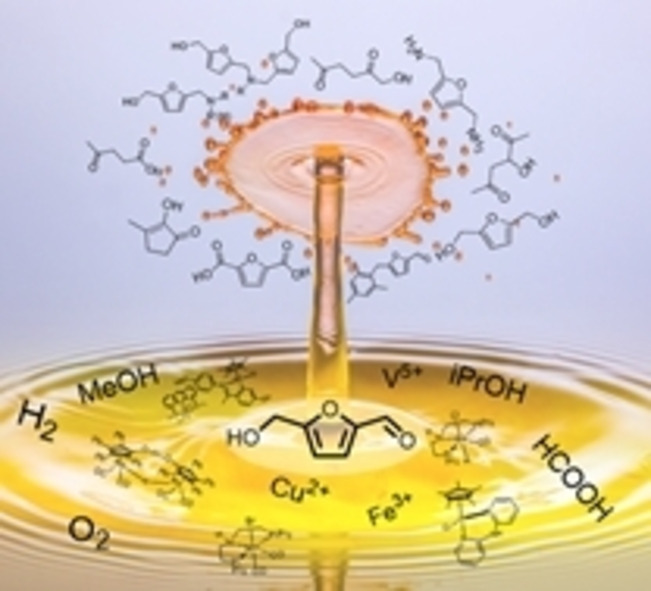

## 1 HMF as a Platform Chemical

**Figure 1:**
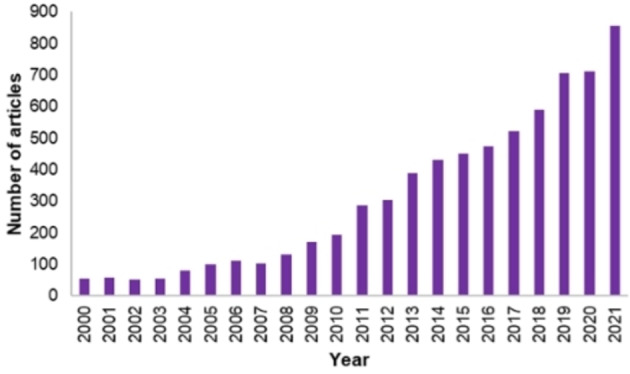
Publications output distribution related to HMF from 2000 to 2021 according to Scopus.

As the worldwide production and utilization of fossil‐based products is continuously rising, there is an urgent need in transferring this production to the side of renewables, employing green resources and energy to obtain the same or analogous products.^[1]^ In this optic, all the possible routes must be passed through selecting them depending on local resources and possibilities. Among these, biomass utilization stands out for its worldwide distribution and high annual production (≈170 billion metric tons), which makes it a unique feedstock.^[2]^ In this framework the conversion of biomasses through “biorefinery” has been introduced. The concept of biorefinery relies on the production of chemical building blocks from biomass that can substitute analogous ones produced from petrol. In fact, because of their complex composition, biomasses allow to obtain a plethora of different molecules, which can be further processed to obtain petrol substitutes. Among the wide pool of these chemicals, 5‐hydroxymethylfurfural (HMF) has enormous potential, as also confirmed by the continuously increasing number of publications in the field in recent years (Figure 1).

**Scheme 1:**
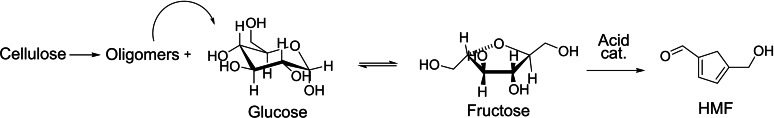
HMF production from cellulose, via glucose and fructose.

To obtain HMF from biomasses, these undergo acid‐catalyzed hydrolysis to hexoses, such as fructose or glucose, which is then followed by dehydration to HMF (Scheme 1). It is worth noting that the latter reaction is more efficient and selective when starting from fructose than glucose.^[3]^ The dehydration of fructose is an acid‐catalyzed reaction. The simplest choice for fructose conversion is using aqueous solutions of mineral acids such as sulfuric acid, hydrochloric acid, and phosphoric acid.[^4^, ^5^] However, the use of specific aprotic organic molecules acting as catalysts and solvents, such as DMSO, can increase yield and selectivity. On the opposite, they pose a less green route for HMF production.^[6]^ Finally, classical acid heterogeneous catalysts are employed. On the other hand, glucose is a preferable feedstock for HMF production because of its lower cost than fructose. Moreover, it is among the most present monosaccharides in biomass and can be easily obtained by acid or enzymatic hydrolysis of cellulose, starch, or sugar. However, it can undergo enolization reaction to a lower extent, making it step‐limited toward HMF production. In fact, glucose transformation to HMF more easily occurs by glucose isomerization to fructose and its dehydration to HMF rather than direct dehydration of glucose. Thus, a catalytic system able to tandemly isomerize glucose to fructose and dehydrate fructose to HMF is needed in this process. Different catalysts and systems can be employed for HMF production from glucose. Among them we can cite metal chlorides (AlCl_3_), Brønsted acids (HCl), Lewis acids (zeolites), or supported catalysts (e. g., Al over MCM‐41)[^7^, ^8^, ^9^, ^10^, ^11^, ^12^] Finally, cellulose is an abundant and widely distributed biomass component that can be used to obtain HMF. It consists of several glucose units linked together in a polymeric structure with 1,4‐glycosidic bonds.^[13]^ The direct conversion of cellulose to HMF instead of passing through the glucose production step is an attractive route, though more challenging. In fact, it involves different consecutive steps such as cellulose hydrolysis into glucose, its isomerization to fructose, and finally the dehydration step of fructose to HMF.^[14]^ For this reason, it has not been still applied to an industrial level.^[15]^ Up to date, cellulose has been converted to HMF using metal chlorides such as FeCl_3_, RuCl_3_, VCl_3_, TiCl_3_, MoCl_3_, and CrCl_3_.^[16]^ An even greener approach to glucose, fructose, and cellulose conversion to HMF and further products, is found in photocatalysis, although HMF productivity is still not competitive with thermochemical processes.[^17^, ^18^, ^19^]

Among other bio‐based building blocks HMF displays an intriguing structure with a furan ring coupled with hydroxide and aldehydic functionalities available for several transformations.^[20]^ For instance, the hydroxide can be esterified, dehydrated, or oxidized. On the other hand, the aldehyde can undergo reduction, decarbonylation, oxidation, or reductive amination. Finally, the furan ring can be transformed with reactions such as halogenation, sulfonation, nitration, Diels‐Alder cycloaddition, and Friedel‐Crafts alkylation or acylation.[^21^, ^22^] In a biorefinery perspective, these products can replace fossil‐derived ones such as alkyldiols, hexamethylendiammine, or adipic acid (applied to polymer production); they can be used in pharmaceutics, textile production, or directly applied as biofuels or solvents.[^22^, ^23^, ^24^]

HMF selective oxidation represents one of the most interesting and variable reactivities of HMF due to the large variety of valuable molecules that can be obtained, such as 2,5‐diformylfuran (DFF), 2,5‐furandicarboxylic acid (FDCA), 5‐formyl‐2‐furan carboxylic acid (FFCA), 5‐hydroxymethyl‐2‐furancarboxylic acid (HMFCA), and maleic anhydride (MA).[^25^, ^26^] DFF is a platform molecule that can be employed as an intermediate for further target molecules.^[27]^ Moreover, it can be directly used as monomer in polymer synthesis. FDCA is an important monomer that has been addressed as a substitute of terephthalic acid for bio‐polymers production and has also been applied as pharmaceutical precursor.^[28]^ FFCA and HMFCA have potential in the polymer and pharmaceutical industry as well.^[29]^ MA is an important building block toward the production of several products such as unsaturated polyester resins, 1,4‐butanediol, fumaric acid, and tetrahydrofuran, and its synthesis from bio‐based substrates is still underdeveloped.^[30]^ Heterogeneous catalysts such as metal oxides promote the formation of DFF in aqueous phase, by favoring the selective oxidation of the alcohol moiety.[^31^, ^32^] On the opposite, the aldehyde can be oxidized to HMFCA or FDCA usually using heterogeneous supported noble metals, basic supports, and basic solvents.[^33^, ^34^, ^35^, ^36^, ^37^, ^38^, ^39^, ^40^, ^41^, ^42^, ^43^]

Another largely studied reactivity regards HMF reduction, which can provide numerous bio‐based intermediates as well, such as 2,5‐bishydroxymethylfuran (BHMF), tetrahydrofuran‐dimethanol (THFDM), 1,6‐hexanediol (1,6‐HD), and 2,5‐dimethylfuran (DMF). BHMF can be used as biodiesel additive, a non‐ionic surfactant, a monomer, or a substrate in the flavor industry, THFDM as a green solvent, while 1,6‐HD is used as a green monomer for polyester and polyurethane production. Finally, DMF is a potential fuel thanks to its high energy density.[^44^, ^45^, ^46^, ^47^]

In general, hydrogenation offers more reaction pathways than oxidation as it can involve the modification of hydroxyl, aldehyde, and furan moieties. Beyond pressurized molecular hydrogen, it can undergo transfer hydrogenation usually employing alcohol or in formic acid as solvent.[^48^, ^49^] Furthermore, HMF reduction can be exploited to produce diketones or ketoacids such as 1‐hydroxyhexane‐2,5‐dione (HHD) and 3‐hydroxyhexane‐2,5‐dione (3‐HHD) from HMF under hydrogenation/hydrolytic ring‐opening conditions. To cite a few, Pt/C, Pd/C, Au/TiO_2_, Rh−Re/SiO_2_, and Au/Nb_2_O_5_ heterogeneous catalysts have been reported for HMF hydrogenation.[^50^, ^51^, ^52^, ^53^, ^54^, ^55^]

On the other hand, levulinic acid (LA), a ketoacid, can be obtained by dehydration of HMF. LA is an intriguing building block that can be used for the synthesis of green chemicals applied as fuel additives, resins, or polymers precursors. It can be easily obtained from HMF using mineral acids such as HCl or H_2_SO_4_. However, these conditions may lead to an abundant formation of polymerization products (humins).^[56]^

Etherification of HMF with aliphatic alcohols allows the production of alkoxymethyl furfurals (AMF).^[57]^ These products, such as 5‐methoxymethyl furfural or 5‐ethoxymethylfurfural, can be employed as fuels additives thanks to their high energy density. The reaction is usually favored over strong acid sites. Weak acid sites favor acetalyzation to 5‐(alkoxymethyl) furfural dialkylacetal (EMFDEA).[^21^, ^58^, ^59^] Aldol condensation is a reaction that allows to produce fuel precursors from short‐carbon‐chain carbonyl or hydroxy‐containing molecules through coupling with HMF. The obtained products can be then easily transformed in fuels with high octane number.^[60]^

Apart from being the base for fuel production and formulation, HMF can be also employed in the formation of building blocks for the pharmaceutical industry. This is the case of *N*‐substituted‐5‐hydroxymethyl‐2‐furfuryl amines. These are the results of reductive amination of the HMF molecule with different amines or ammonia in a reductive atmosphere (pressurized H_2_) or by reaction with nitrobenzene in the presence of, to cite an example, a heterogenous Pd/C catalyst.[^61^, ^62^]

Another pharmaceutical precursor that can be obtained from HMF is furfuryl alcohol (FA). Interestingly, this molecule also finds applications in polymers, the foundry industry, and can be widely employed as solvent. Furfuryl alcohol is traditionally produced from the corresponding aldehyde (i. e., furfural) being the target of 65 % of its global production.^[63]^ Nevertheless, HMF decarbonylation can directly lead to furfuryl alcohol showing an alternative route for its obtainment from renewables.[^64^, ^65^]

**Figure 2:**
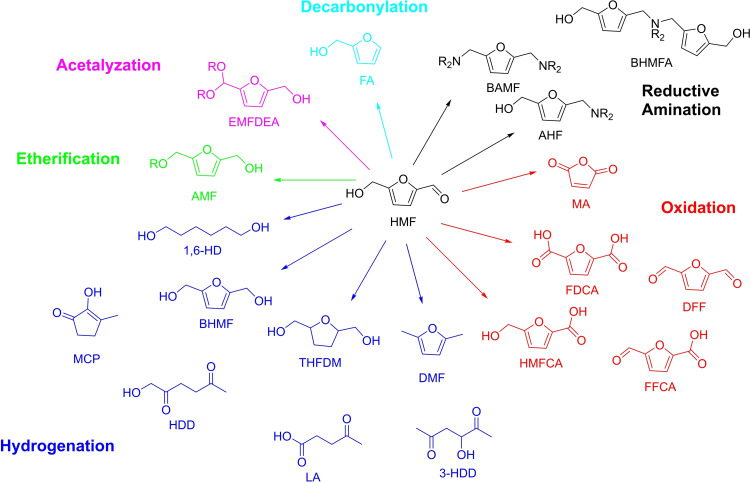
Bio‐based platform products that can be obtained from HMF.

Other products can be obtained from HMF using different production processes. However, a complete description of all the possible conversion methods for HMF is beyond the scope of this work and has been reported elsewhere.^[66]^ Nevertheless, the considerable number of reactions and products that can be obtained from HMF (Figure 2) can be concurrently seen as a challenge and an opportunity. In fact, to discriminate between this wide pool of reactions and selectively activate the desired functional group, there is a need for developing active and selective catalysts together with screening of reaction conditions and reaction environment.^[67]^

This aim could be achieved with the complementary development of homogeneous approaches in which molecular catalysts allow a finer tuning by design of ligands and corresponding complexes. In general, although classical conversion of HMF is carried out by heterogeneous catalysts, homogeneous processes have been recently introduced, aiming at the improvement of reaction selectivity and at energy saving by means of milder conditions. An intriguing advantage of this complementary approach is also represented by the easier interpretation of mechanistic insights. In‐situ characterization available in the liquid phase, typical of these approaches, helps to figure out useful information to be implemented in all branches of catalysis.

The first homogeneous catalytic system for HMF transformation appeared in 2001 when Partenheimer and Grushin^[68]^ exploited dioxygen and metal/bromide catalysts (e. g., Co/Mn/Zr/Br) for the synthesis of DFF and FDCA, which were isolated in 57 and 60 % yield, respectively. Although unexpected for that time, low selectivity was obtained, and quite harsh conditions were needed (e. g., 70 bar of air at 75 °C for DFF synthesis and at 125 °C for FDCA). Following this early result, the study of HMF as a suitable substrate in homogeneous catalysis has been underdeveloped until 2008 when a transition metal molecular catalyst (namely a Mn^III^‐salen catalyst) allowed a higher‐yielding approach for oxidation of HMF to DFF.^[69]^ The reaction occurred at room temperature in a biphasic system (pH 11.3 from phosphate buffer in CH_2_Cl_2_, catalyst loading of 5 mol%) in the presence of sodium hypochlorite as the primary oxidant with yields up to 89 %. Although still not environmentally benign due to the use of dichloromethane, this example paved the way to the flourishing of studies of the last decade, during which several publications demonstrated that the majority of the HMF transformations traditionally mediated by heterogeneous catalysis could be reproduced under homogeneous liquid‐phase conditions. HMF transformations such as oxidations, hydrogenations with molecular H_2_ or hydrogen transfers, hydrogenation/hydrolysis to diketone and ketoacids, reductive aminations, decarbonylation, and Friedel‐Craft arylation can be controlled by the appropriate combination of a soluble transition metal source (assisted by added ligands and co‐catalysts) or by the rational design of a molecular catalyst. The aim of the present Review is to report the advances in homogeneous catalysis in HMF conversion with a critical analysis of its lights and shadows. A special focus is given to the description of catalytic systems, the role of ligands and metals, co‐catalysts, mechanistic insights, and tuning of reaction efficiency where available.

## 2 HMF Oxidation to DFF, MA, or FDCA

**Scheme 2:**
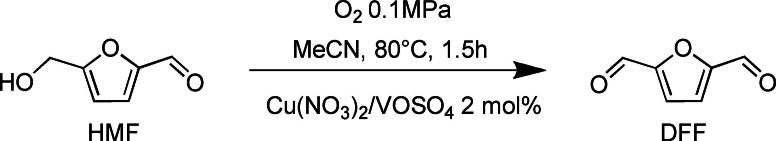
Catalytic oxidation of HMF to DFF.^[70]^

Starting from what was previously described, it becomes important from both an economic and environmental point of view to avoid the use of toxic stoichiometric oxidants such as MnO_4_^−^ and Cr_2_O_7_^2−^ for the oxidation of HMF. Research was thus dedicated from now on to the use of molecular oxygen as terminal oxidant. However, owing to the presence of both a furan ring and an aldehyde group in the molecule, the oxidation of HMF to DFF often involves many side reactions, such as overoxidation, decarbonylation, and cross‐polymerization. Thus, selectivity to DFF remains the greater challenge and it can be reached only by employing more efficient catalytic systems, possibly under milder conditions. In 2011, Pang and co‐workers^[70]^ reported on a homogeneous catalytic system, which combined Cu(NO_3_)_2_ and VOSO_4_, that was able to oxidize HMF to DFF in a facile manner with good yields under mild conditions (Scheme 2).

**Scheme 3:**
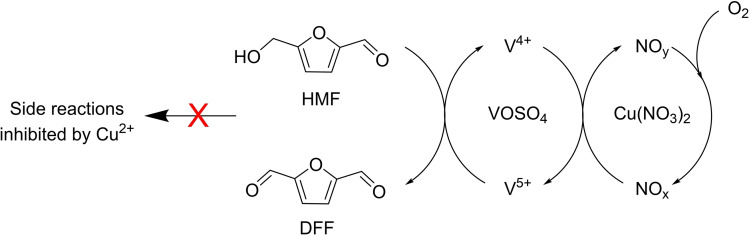
Aerobic oxidation of HMF to DFF promoted by Cu(NO_3_)_2_ and catalyzed by VOSO_4_.^[71]^

Cu(NO_3_)_2_ is a strong oxidant and was exploited as assistant to the V^V^ species during oxidation with molecular oxygen. An HMF conversion of up to 99 %, with 99 % DFF selectivity, was obtained in 1.5 h at 80 °C in acetonitrile, with a catalyst loading [Cu(NO_3_)_2_/VOSO_4_ in 1 : 1 molar ratio] of 2 mol% and 0.1 MPa O_2_. Interestingly, although needing more time, the oxidation even proceeded at room temperature (48 h). Finally, the inorganic catalyst was claimed to be easily removed after oxidation. Unfortunately, no data on the use of the recovered catalyst and its stability were provided. Further insights on this catalytic system have been implemented by the same group in 2014,^[71]^ demonstrating the peculiar role of Cu^2+^ on the selectivity to DFF, namely in preventing the oxidative C−C bond cleavage of HMF and radical reactions of DFF itself to polymerized compounds (humins). In‐situ Fourier‐transform infrared (FTIR) spectroscopy further demonstrated that Cu(NO_3_)_2_ is necessary to transform VOSO_4_ into active V^V^ species, and this process occurs through the generation of NO_*x*_ gas. On the opposite, other metal nitrates such as Ce(NO_3_)_3_, Ni(NO_3_)_2_, Mn(NO_3_)_2_, Co(NO_3_)_2_, NaNO_3_, or Fe(NO_3_)_3_, not able to degrade to NO_*x*_, showed by far lower activity. The effect of solvent polarity was also established, showing that acetonitrile performed best. In addition, kinetic studies finally led to the establishment of the reaction mechanism reported in Scheme 3.

The effect of O_2_ pressure on the HMF conversion and DFF selectivity was examined and was found to be low as the reaction was not controlled by an oxygen mass‐transfer process.

A significantly different behavior was found by the same group when VO(acac)_2_ was employed at higher oxygen pressures as a catalyst in the liquid‐phase oxidation of HMF to MA with molecular oxygen.^[72]^ VO(acac)_2_ was found to be active [as was the case for the Cu(NO_3_)_2_/VOSO_4_ system]^[70]^ in the formation of DFF at 0.1 MPa of O_2_, but up to 52 % yield of MA was obtained upon increasing oxygen pressure.[^73^, ^74^]

**Scheme 4:**
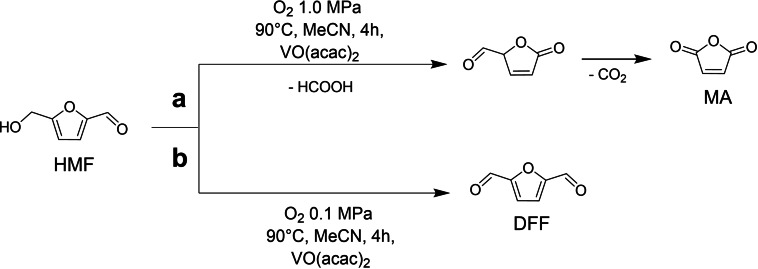
VO(acac)_2_‐catalyzed HMF oxidation pathways to MA (route a) and DFF (route b).^[72]^

Best results were achieved in acetonitrile or acetic acid leading to quantitative conversion of HMF at 90 °C at 1.0 MPa O_2_. Anyway, the obtained yield was found to be around 50 %, which needs to be increased especially with regard to industrial application. The good performances of VO(acac)_2_ encouraged authors to perform several experiments to propose the reaction pathway (Scheme 4). The results showed that C−C bond cleavage occurred, under 1.0 MPa O_2_ atmosphere, simultaneously to the oxidation on the hydroxymethyl group of HMF toward the formation of MA (Scheme 4a), while upon lowering O_2_ pressure to 0.1 MPa O_2_, the oxidation of HMF to DFF is favored (Scheme 4b).

**Figure 3:**
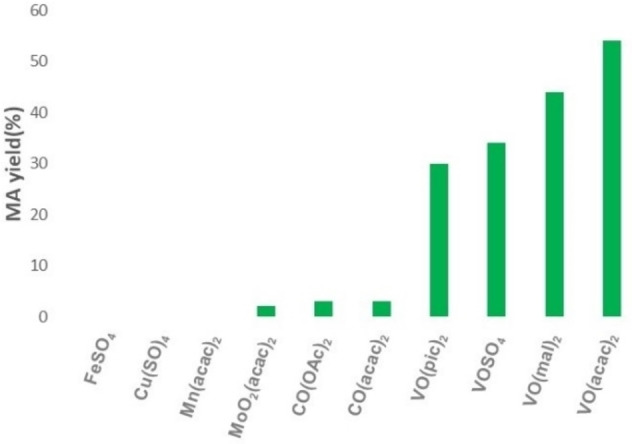
Oxidation of HMF to MA catalyzed by different transition metals.

The results obtained are quite intriguing as the product selectivity could be easily and effectively tuned by a simple modification of the reaction conditions. The uniqueness of vanadium species for catalytic C−C bond cleavage has been proved by comparison with vanadium complexes and several other first‐row transition metals such as FeSO_4_, CuSO_4_, Mn(acac)_2_, MoO_2_(acac)_2_, Co(acac)_2_, and Co(OAc)_2_. Indeed, the latter were significantly less effective (Figure 3).

**Scheme 5:**
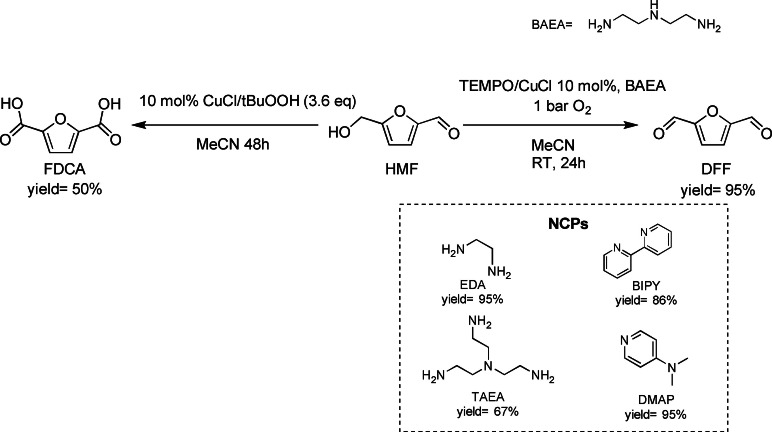
Cu‐catalyzed oxidation of HMF to FDCA and DFF.^[75]^

Going back to efficient transformation of HMF to DFF with air/O_2_, the oxidizing system consisting of the radical 2,2,6,6‐tetramethyl‐piperidin‐1‐oxyl (TEMPO), CuCl, and a nitrogen‐containing promoter (NCP) was later deeply studied by Riisager and co‐workers.^[75]^ They took inspiration from the catalytic system designed in a previous work by Cottier et al.^[76]^ Screening of the solvents, reaction conditions, and several chelating ligands such as *N*,*N*‐dimethylaminopyridine (DMAP), 2,2’‐bipyridine (bipy), ethylendiammine (EDA), bis(2‐aminoethylamine) (BAEA), and tris(2‐aminoethylamine) (TAEA) clearly demonstrated the fundamental role of NCPs. Oxidation of HMF with CuCl/TEMPO catalytic system in MeCN using dioxygen showed a high selectivity to DFF (97 %) but low conversion (44 %). Nevertheless, the addition of several NCPs (up to 1 : 1 ratio with CuCl) seriously boosted the conversion at unaltered selectivity. Up to 95 % DFF yield was obtained with high selectivity in 24 h in MeCN at room temperature, with a CuCl/TEMPO loading of 10 % and atmospheric pressure of O_2_ in presence of BAEA ligand (Scheme 5).

Although the influence of the NCPs ligands on CuCl behavior has not been completely explained yet, it is clear that they play a crucial role in HMF conversion. Nevertheless, they also promote the catalytic activity in other solvents than MeCN, enlarging the field of application. One example is methyl isobutyl ketone (MIBK), usually employed as a green solvent for HMF extraction in its synthesis from fructose. This solvent can thus be imagined suitable for multistep reactions.

The CuCl‐based catalyst (10 mol%), when paired with *tert*‐butyl peroxide (*t*‐BuOOH, 3.6 equiv.), was also able to further oxidize HMF into the renewable terephthalic acid substitute FDCA in fair yields (50 %) in MeCN at room temperature in 48 h (Scheme 4). Interestingly, the product was easily separated by simple filtration. These results are interesting when compared with those provided by heterogeneous catalytic systems, in which very high pressures and temperatures are required. This approach thus represents a mild alternative employing non‐expensive metals. There is still the need to improve the conditions and catalyst recyclability to make the reaction available for real applications.

**Scheme 6:**
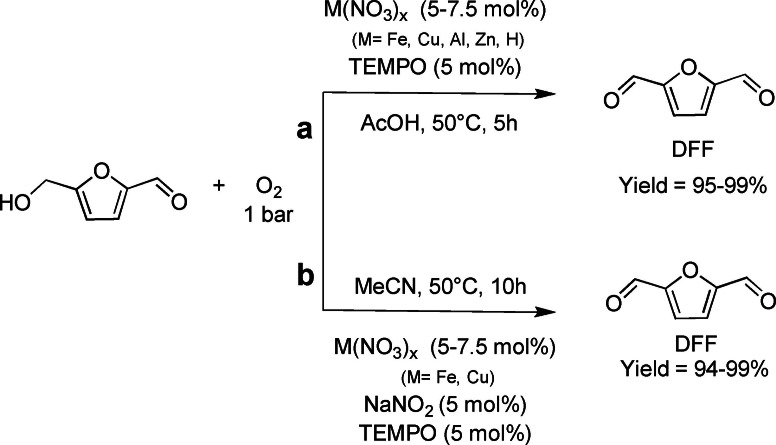
HMF oxidation to DFF with metal nitrates and TEMPO.^[77]^

The peculiar reactivity of TEMPO as co‐catalyst for the oxidation of HMF to DFF under aerobic conditions has been also very recently explored by Hong et al.^[77]^ in combination with metal nitrate based on Earth‐abundant metals. Using cost‐beneficial M(NO_3_)_*x*_/TEMPO (M=Fe, Cu, Al, Zn) systems, reaction parameters such as metal catalysts, catalyst loading, solvents, and temperature were screened employing acetic acid or acetonitrile, respectively, working with a TEMPO loading of 5 %, a metal catalyst loading of 5–7.5 % at 50 °C in 5 h under atmospheric pressure (Scheme 6a).

Best catalytic efficiency (i. e., quantitative conversion of HMF and high selectivity in DFF) was obtained with Fe(NO_3_)_3_ and Al(NO_3_)_3_ at 5 mol%. For Zn(NO_3_)_2_ and Cu(NO_3_)_2_ a slightly higher catalytic loading was needed (7.5 mol%) in glacial acetic acid to achieve similar performances. These results together with the lower efficiency of HNO_3_ and iron salts with different counterions [e. g., Fe(acac)_3_, FeCl_3_] are interpreted taking in account the fundamental role of NO_3_^−^ anions and their quantity in the reaction environment. Furthermore, the activity of nitrates with redox‐inactive metals such as Al(NO_3_)_3_⋅and Zn(NO_3_)_3_ demonstrated that the oxidation of HMF does not involve a redox change at the metal site. Anyway, the cation must be involved, probably due to interaction with TEMPO, because alkaline salts are far less active than M^2+^ and M^3+^ metals. Moving to more eco‐friendly and economic air instead of O_2_ with Fe(NO_3_)_3_ still gives satisfying results (HMF conversion 82 %, selectivity 99 %) under the same reaction conditions.

**Scheme 7:**
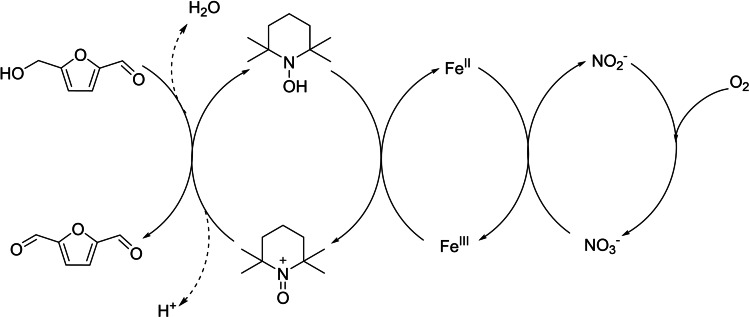
HMF oxidation mechanism in presence of metal nitrates and TEMPO.^[77]^

The catalytic system proved to perform at a slower rate in acetonitrile compared to acetic acid. Thus, to improve the efficiency in the former, NaNO_2_ was implemented as an additive (5 mol%). This allowed the new catalytic systems M(NO_3_)_*x*_/TEMPO/NaNO_2_ (M=Fe, Cu) to reach complete conversion in 10 h (Scheme 6b). Noteworthy, these improvements in the reaction outcome in CH_3_CN occurred only in the presence of redox‐active metals such as Fe and Cu, allowing the depiction of a plausible reaction pathway (Scheme 7).

The solvent effect of the new systems and Fe(NO_3_)_3_/TEMPO/NaNO_2_ (M=Fe, Cu) was further examined, and the more industrially friendly solvent ethyl acetate (EtOAc) was found to maintain the same efficiency under the same conditions.

These examples represent a step forward toward the discovery of cheaper, milder, and greener methodologies by designing inexpensive catalytic systems for selective aerobic oxidation of HMF to DFF.

Nevertheless, homogeneous catalysis is usually held back by issues in the purification of catalyst and products. A modern and elegant way to immobilize homogeneous catalysts for recovery and recycling is the use of ionic liquids (ILs) as solvents or for biphasic mixtures. Over the past decade, ILs have gained wide recognition in the field of green synthesis, where they can act as environmentally benign solvents and support various chemical processes, including catalysis.[^78^, ^79^, ^80^] In 2020, taking advantage of the work by Riisager and co‐workers,^[75]^ Hallett and co‐workers^[81]^ extended the oxidation of HMF to DFF using oxygen (1 atm) with a TEMPO/CuCl catalyst system and a range of imidazolium‐based ILs as solvents in the presence of a base at different HMF substrate loadings (10–50 %). ILs could overcome drawbacks derived from the toxicity of acetonitrile, separation and purification problems (e. g., TEMPO removal), low substrate loadings, or high oxygen pressures. ILs have low volatility and potential for recyclability, which has led to their widespread use in biomass processing, such as the conversion of fructose, glucose, and cellulose to HMF in these media.[^67^, ^82^, ^83^] Despite these advantages, the isolation of HMF from IL medium is still a challenge. On the other hand, the conversion of HMF to DFF in ILs is particularly attractive due to the ability of DFF to sublime from the IL. The study of four different ILs (1‐butyl‐3‐methylimidazolium [bmim]^+^ with several counterions such as chloride, bromide, protic/noncoordinating, and noncoordinating anions) displays a dramatic difference in the conversion of HMF to DFF. The CuCl/TEMPO system showed best performances in the nonprotic, noncoordinating ILs {i. e., 1‐butyl‐3‐methylimidazolium triflate [bmim][OTf] and 1‐butyl‐3‐methylimidazolium bis(trifluoromethylsulfonyl)imide [bmim][NTf_2_]} in the presence of a nitrogen base such as pyridine, yielding up to 90 % DFF (5 mol% catalyst loading, 12 h, 80 °C, O_2_ atmosphere). Good selectivity for DFF was observed over the product of further oxidation (FFCA), even at high substrate loadings (50 wt%). Noteworthy, high substrate loadings and no need for high oxygen pressures are improvements compared with the typical conditions for processes based on heterogeneous catalysis.

**Scheme 8:**
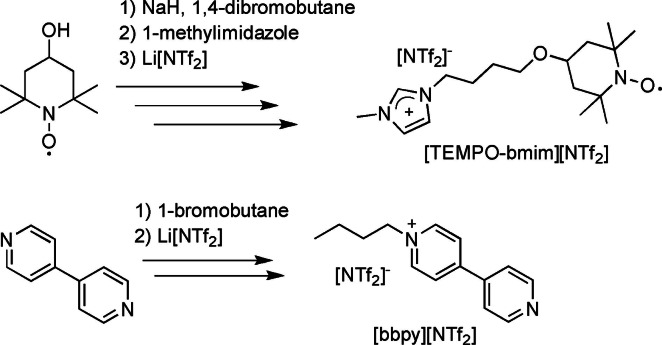
Synthesis of TEMPO and pyridine ionic liquids.^[81]^

A great challenge is still found in the recyclability of the system, and further studies should be focused on this topic. From this point of view, improvement of the system efficiency was implemented immobilizing both TEMPO and pyridine on the IL (Scheme 8).

The use of this supported TEMPO system in [bmim][NTf_2_], prevented the sublimation of TEMPO during the purification DFF, promoting high isolated yields (81 %; HPLC yield 91 %). Furthermore, introduction of immobilized TEMPO and pyridine components allowed recycling of the catalytic system after sublimation of the DFF product. The lower conversion of 75 % and yield of 68 % that were obtained in the second cycle confirm that the recycling of homogeneous catalysts remains one of the key challenges in this field.

Evolution of the results described for HMF oxidation to DFF well represents the potential role of homogeneous catalysis in solving problems related to selectivity and energetic cost of the process. Thanks to rational design, the research process did one step after another, finding ways to improve efficiency of catalytic systems and in 2020 a first way to recycle. There is plenty of room then to also solve this problem by designing efficient and stable catalysts to scale up the process and to achieve economic feasibility.

A renewed interest has recently been focused on the optimization of homogeneous oxidation of HMF to FDCA with the Partenheimer/Grushin Co/Mn/Br system^[68]^ by Zuo et al.^[84]^ which improved FDCA yield to about 90 %, and studied the reaction kinetics including gas‐liquid mass transfer.^[85]^ An accurate and comprehensive screening of the oxidation system was further developed by Cheng et al. in 2020,^[86]^ leading to a rapid and highly selective oxidation of HMF to FDCA in acetic acid (HAc) with air as the oxidant and Co/Mn/Br catalyst. Careful and critical evaluation of the main and side reactions upon changing temperature, pressure, substrate, solvent ratio, catalyst composition, and concentration nicely allowed to reduce the side reactions (HMF overoxidation to CO and CO_2_, HMF condensation to polymers, etc.). Optimal conditions, namely 150 °C, 0.8 MPa of air pressure, a 29 : 1 : 30 molar ratio of Co/Mn/Br, and a high mass ratio of HMF/HAc (1 : 9), increased selectivity of FDCA to 92 % (purity >99.8 %) with total conversion of HMF in less than 20 min. This results make the homogeneous system promising for the optimization of HMF oxidation and for the large‐scale production of FDCA, with a process inspired by the Co/Mn/Br AMOCO midcentury (MC) catalyst.^[87]^ This general reaction network would lead to a more feasible FDCA production method than those available for heterogeneous systems, since it overcomes the separation problems of solid FDCA. Here, it is recovered by crystallization, filtration, and drying, leaving the dissolved Co/Mn/Br catalytic system available for recycling.

## 3 HMF Reduction

### 3.1 HMF reduction with molecular H_2_

**Scheme 9:**
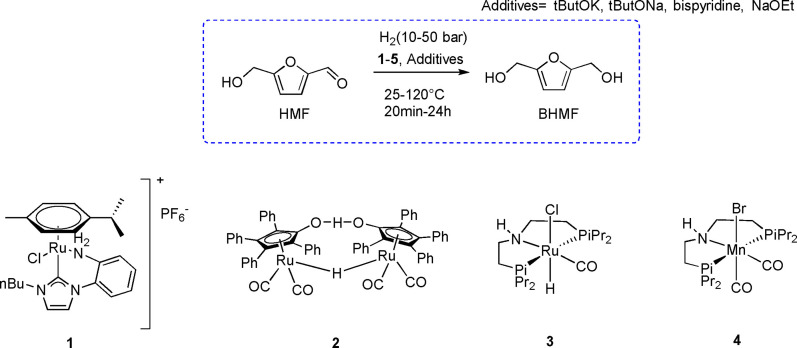
HMF reduction with molecular hydrogen and pre‐catalysts **1**–**4** described in the following.[^89^, ^98^, ^100^, ^101^]

As previously introduced, the hydrogenation of HMF to BHMF is an industrially relevant reaction allowing the formation of an important building block in polymer and flavor industry. Generally, this reaction is conducted using heterogeneous systems where high temperatures (140–200 °C) and pressures (70–75 bar) are required; conditions that commonly affect reaction selectivity. Selective homogeneous reduction of HMF to BHMF requires the employment of bifunctional catalysts, bearing a base in the molecular structure, namely on one of the ligands, and an available site to form a metal hydride during the catalytic cycle. Within this field, rational design of the molecular pre‐catalysts becomes fundamental to address the selective reduction of the aldehyde instead of other reactive functional groups such as furanic double bonds and the alcoholic moiety. In Scheme 9, pre‐catalysts classically employed for this reaction are reported. Ruthenium molecular pre‐catalyst **1** was firstly explored by Elsevier and co‐workers^[88]^ in 2013. The homogeneous catalyst (0.5 mol% loading) quantitatively converted HMF into BHMF in 2 h at 70 °C and 50 bar of H_2_. **1** is composed by an amino group stabilized by an N‐heterocyclic carbene (NHC) ligand combined with an halogenide combination that favors the bifunctional hydrogenation process.^[89]^ The authors found that catalytic performances are strongly dependent on steric effects of the chelating ring and amine basicity. A shorter induction time was observed with small, strained chelating rings and less basic benzyl amines that favored substrate coordination.

**Scheme 10:**
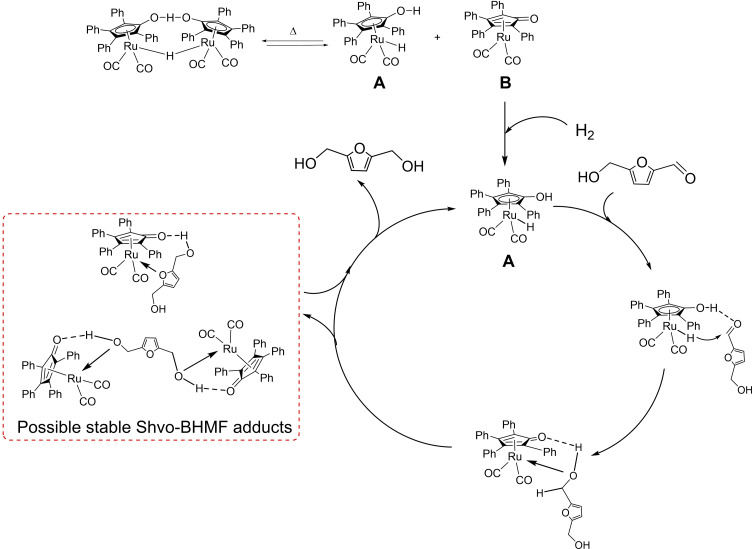
Mechanism for the reduction of aldehydes mediated by Shvo **2** catalyst.^[98]^ The latter activates molecular hydrogen toward the formation of complex **A**. Then, the aldehyde approaches the catalyst in the outer sphere, and the reduction occurs due to the concerted transfer of hydride and acid hydrogen on the aldehyde, closing the catalytic cycle. This concerted mechanism allows mild reaction condition and high selectivity toward the reduction of the sole aldehyde moiety. Best conditions were catalyst loading of 0.1 mol% in toluene at 90 °C and 10 bar of H_2_ pressure, where BHMF was obtained quantitatively after just 1 h.

Ru‐hydride‐containing Shvo catalyst **2** exploits the cyclopentadienone ligand as the basic site for the selective bifunctional hydrogenation of polar double bonds[^90^, ^91^, ^92^, ^93^, ^94^, ^95^, ^96^, ^97^] and proved very useful also in HMF selective reduction of BHMF.^[98]^ Noteworthy, the complex **2** is not the main actor of catalysis but behaves as catalyst precursor, leading to active species that participate in the HMF conversion mechanism. In fact, it can be split, after heating, into **A** and **B** species (Scheme 10).

**Figure 4:**
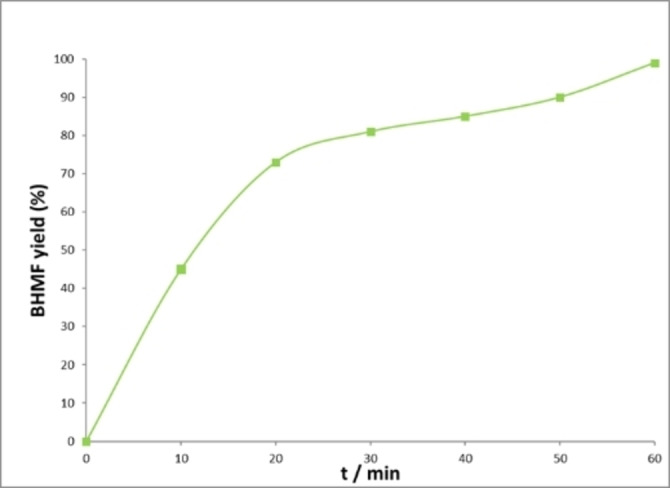
BHMF yield over time observed with Shvo catalyst.^[98]^

A kinetic study unfolded a peculiar behavior on HMF conversion over time that showed a slowdown in conversion after reaching 70–90 % conversion (Figure 4).

This behavior was attributed to an inhibition effect exerted by the reagent HMF and the product BHMF, which form stable adducts with the catalyst as demonstrated by in‐situ NMR spectroscopy and density functional theory (DFT) calculations (Scheme 10, red box).

The optimized conditions reported above were chosen to study recyclability and re‐use of the catalyst employing two different methods: product extraction with water, and BHMF precipitation and removal by filtration. In the first method, at the end of the reaction the toluene solution was washed with water to collect BHMF, and the organic catalytic solution was reused. However, water‐soluble ruthenium intermediates were transferred into the aqueous solution and partial complex decomposition occurred. On the other hand, best efficiency was reached exploiting BHMF solubility in toluene, which decreased upon cooling down the reaction mixture. BHMF precipitated quantitatively, allowing its collection by a simple filtration at the end of every cycle. Nicely, this second recycle method preserved the catalytic activity up to nine cycles.

**Scheme 11:**
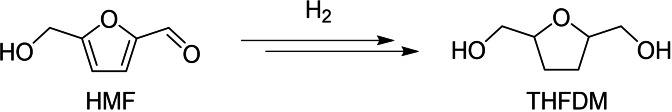
General scheme for HMF reduction to THFDM.

In the previously described works, reduction of HMF to BHMF was achieved avoiding over‐reduction to unwanted side products such as THFDM (Scheme 11).

**Scheme 12:**
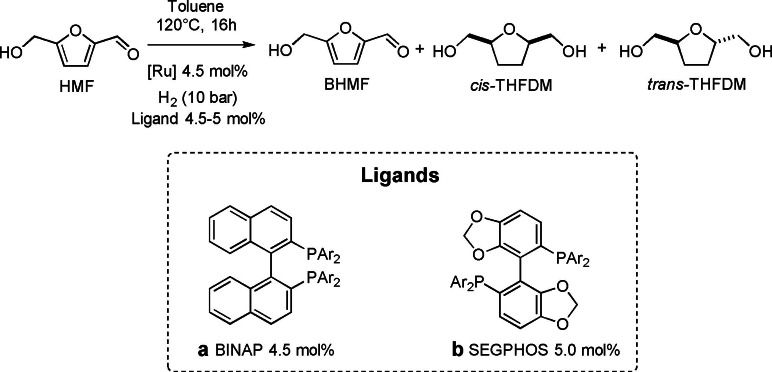
Ruthenium‐catalyzed HMF reduction to THFDM.^[99]^

This product, however, represents a useful bio‐based substrate for the production of polyurethane and polyesters as previously introduced. Hashmi and co‐workers started to investigate the possibility of obtaining THDMF from HMF using homogeneous systems^[99]^ with a particular focus on stereoselectivity. This is a nice example of catalyst design by combinatorial chemistry. Various catalytic systems based on ruthenium, iridium, and rhenium combined with different pre‐ligands were tested. At first, the reaction was conducted using Ru(methylallyl)_2_COD complex (4.5 mol%) in the presence of potassium *tert*‐butoxide (13.5 mol%) and variously substituted imidazolium salts as NHC pre‐ligands (9 mol%). Unsaturated and aromatic backbones proved to be more active than the saturated ones in the hydrogenation but, in general, low *cis*/*trans* selectivity was achieved (1.26 : 1). Screening different rhodium, ruthenium, and iridium hydrogenation pre‐catalysts allowed to tune the selectivity of the system. Results generally suggest that diphosphine or diphosphate are viable ligands to increase *cis*/*trans* ratio in THFDM (Scheme 12). It appeared that bulkier aromatic ligands were the most active and selective. In particular, the smaller the bite angle and the less flexible the backbone chain, the higher conversion and selectivity was achieved. Best additive ligand was found to be DTBM‐SEGPHOS (Scheme 12, ligand b) that, with a catalyst loading of 4.5 mol%, achieved complete conversion of HMF, a BHMF yield of 3 %, a THFDM yield of 87 %, and a *cis*/*trans* ratio 4.7 : 1 in toluene at 120 °C, 10 atm H_2_, which are milder conditions than for heterogeneous systems. From a tunability point of view, it is nice to observe that a simple change in the backbonding of the chelating phosphine ligand (BINAP; Scheme 12, ligand a) reverses the selectivity at the intermediate BHMF (yield=71 %), THFDM (yield=28 %) under the same conditions.

Moreover, the authors explored the possibility of substituting ruthenium with nickel as a cheaper alternative. Ni(COD)_2_ (4.5 mol%) in presence of BINAP (5 mol%) interestingly displayed similar activity and selectivity to its ruthenium homologue. Among noble metals, iridium and rhodium complexes reported HMF reduction to BHMF, while rhenium reported no activity. To reach complete selectivity in BHMF, nitrogen‐containing ligands were tested with ruthenium as metallic site. Electron‐rich diamine like 2‐dimethylamino‐ethylamine (4.5 mol%) indeed avoided the subsequent aromatic ring reduction, enhancing the hydrogenation chemoselectivity toward the quantitative formation of BHMF.

More recently, Nielsen and co‐workers^[100]^ introduced the application of pincer PNP ruthenium and iridium complexes to the reduction of HMF. Ruthenium complex **3** (Scheme 9) proved to be the most active; with a loading of 0.05 mol%, 2 mol% of NaOEt, 10 bar of H_2_ at 25 °C it led to a 95 % conversion after 15 min and a complete selectivity in BHMF in ethanol. The reaction was faster than those previously reported and occurred at room temperature. However, the presence of sodium ethoxide was essential in the activation of the complex. HMF concentration appeared to influence catalytic performances as well. In fact, a highly concentrated substrate hampered the reaction, while increased hydrogen pressure sped up the kinetics, reaching at 30 bar high conversion in 1 min [turnover frequency (TOF) >1900 min^−1^]. The authors then scaled up the process (1 g of HMF with 100 ppm catalyst loading at 25 °C and 30 bar hydrogen), and after 2 h it was possible to isolate a quantitative yield of BHMF by means of filtration through silica gel. Subsequently, screening reactions in water and water/ethanol mixtures verified the tolerance of the system toward different bases, with LiOH as the optimal one. The recycling of complex **3** was attempted (initial 0.05 mol% of catalyst, 30 bar H_2_, 25 °C, 2 h per run), with consecutive addition of substrate after every cycle affording 75 % conversion after the 3rd cycle. ^1^H NMR spectroscopy and electron spray ionization (ESI) analysis on the reaction crude evidenced the formation of an alkoxide species (Ru‐OR) potentially corresponding to a deactivating Ru‐BHMF species.^[98]^

Regardless of the interesting results that have been reported, the application of homogeneous catalysts in this field has still to be deeply studied, with only few examples reported so far. Anyway, the direction is already well defined. Bifunctional catalysts appear to be the right choice due to lower temperature, pressure, and higher selectivity needed when compared with heterogeneous catalysts. The efficient recycling of some catalysts has also been demonstrated. What remains unsolved from an industrial application point of view is the catalyst cost and the metal employed. From a green perspective, the use of non‐critical raw materials is highly desirable. Following this concept, the first example of a well‐defined first‐row transition metal complex applied in the homogeneous hydrogenation of HMF was reported by Beller and co‐workers.^[101]^ The group described the application of PNP pincer manganese complexes as catalysts in the hydrogenation of polar double bonds. The most active complex **4** (Scheme 9) was tested with different substrates, demonstrating good selectivity and tolerability on different functional groups. The system was applied on α,β‐unsaturated aldehydes under mild conditions (10 bar H_2_, 60 °C), and HMF was converted to BHMF with a 90 % yield (67 % isolated yield). Mechanistic investigation conducted with NMR spectroscopy and supported by DFT calculations reported that, as in the case of ruthenium, the complex underwent the formation of a metal‐hydride species that, in an outer‐sphere mechanism, transferred the hydride and the proton from the metal center to the unsaturated substrate.

### 3.2 HMF reduction to BHMF by transfer hydrogenation

Although hydrogenation with molecular catalysts avoids harsh operative conditions usually required by heterogeneous systems, the use of high pressure of hydrogen is still needed in many hydrogenation processes. One alternative to the use of pressurized gas is found in transfer hydrogenation (TH). This reaction consists of the oxidation of an organic hydrogen source (e. g., isopropanol) that transfers via a mediator a proton and a hydride to a substrate. In this way, polar double bonds, such as aldehydes and ketones, can be reduced to alcohols while avoiding the use of hazardous pressurized gases or stoichiometric reagents like NaBH_4_.

**Scheme 13:**
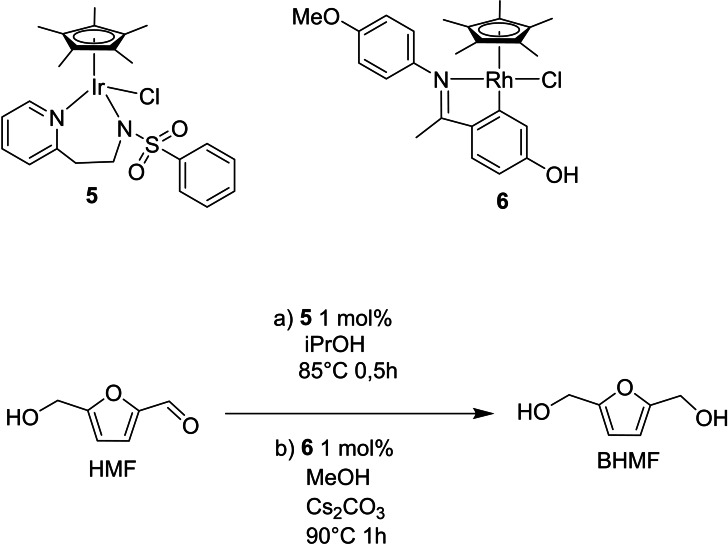
Catalytic HMF reduction by H‐transfer.[^102^, ^103^]

As reported by O'Conner and co‐workers,^[102]^ this reaction usually requires the presence of a base, which, however, can reduce the overall selectivity, facilitating side reactions like aldol condensation. For this reason, the same authors started to investigate Cp*Ir(pyridinesulfonamide)Cl complexes as TH pre‐catalysts for aldehyde reduction in base‐free conditions. Modification of ligands allowed obtaining complexes with different electronic and steric properties. The most active complex **5** was tested in the reduction of HMF to BHMF using 2‐propanol as hydrogen donor. The reaction achieved complete conversion using 1 mol% catalytic loading at 85 °C in 0.5 h, reaching a total BHMF selectivity thanks to the base‐free conditions. So far, this is the only homogeneous example of TH of HMF in absence of base, and it follows a cooperative mechanism (Scheme 13, conditions a).

**Scheme 14:**
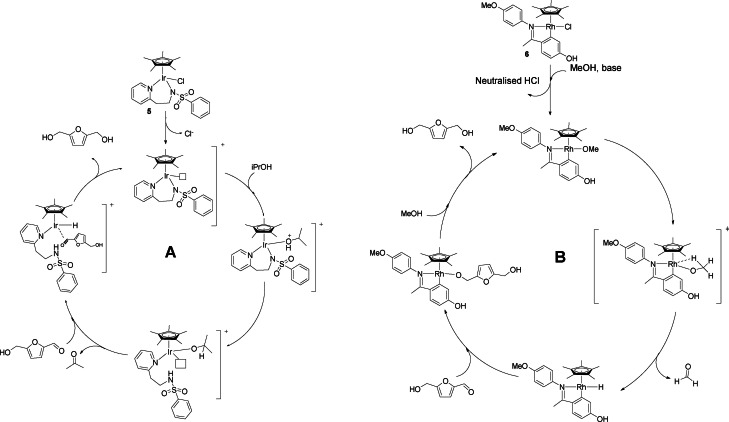
Proposed mechanisms for the Ir (**5**) (cycle A) and Rh (**6**) (cycle B) mediated reduction of HMF to BHMF under homogeneous TH conditions.[^102^, ^103^]

The nitrogen chelating ligand is a hemilabile Lewis base that plays the role of the Brønsted base in the hydrogen abstraction from 2‐propanol. The metal, remaining with a vacant site, receives the hydride from the alkoxide. The cooperative work of metal and ligand is another example of bifunctional catalysis, this time presenting an inner‐sphere mechanism. Except for the activation of the hydrogen source, the remaining step of the cycle retraces the mechanisms described above for the reduction with molecular hydrogen (Scheme 14, catalytic cycle A).

Instead of 2‐propanol, the use of methanol as hydrogen source in TH is highly desirable because it is a bio‐derived, cheap, and abundant alcohol. However, thermodynamics disfavor the reaction with this alcohol, making its use more challenging. Xiao and co‐workers^[103]^ in 2018 started to apply TH to a series of aromatic aldehydes using methanol as hydrogen source and cyclometallate rhodium complexes. In particular, the complex **6** was tested in the TH of HMF, obtaining 82 % yield with a 1 mol% loading and 0.5 equiv. of Cs_2_CO_3_ at 90 °C for 1 h. (Scheme 13, conditions b)

This is a unique example of homogeneously catalyzed TH reduction of HMF with methanol. Conditions are milder than those employed for the same transformations in heterogeneous catalysis, which require higher temperatures (160–320 °C) and typically result in low yields.

In this case, the proposed catalytic cycle (Scheme 14, cycle B) does not withstand a cooperation between a ligand and the metal center. Firstly, the base favors the chloride abstraction while, after deprotonation, MeOH coordinates to the metal as a rhodium‐methoxide. This undergoes β‐hydrogen elimination leading to the Rh−H hydride species. Later, the aldehyde receives the hydride from the metal, and a new Rh‐OR is formed. A hydrogen exchange between MeOH and the Rh‐OR releases the alcohol (ROH, i. e., BHMF), closing the catalytic cycle.

Homogeneous TH applied to HMF is still underdeveloped and deserves to be further investigated due to promising results obtained for both catalytic activity and hydrogen source employed, especially for methanol, which can be obtained from sustainable bio‐resources and used under milder conditions than for heterogeneous systems.

### 3.3 HMF reduction in acidic water to ketoacids and diketones

**Scheme 15:**
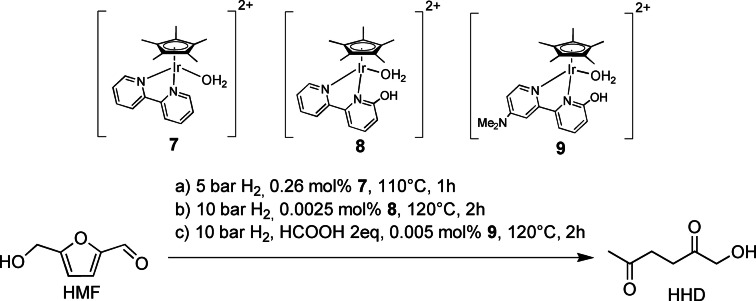
HMF reduction in acidic water to diketones catalyzed by Zhang's iridium complexes **7**–**9**.[^104^, ^105^]

As previously introduced, diketones (e. g., HHD) and ketoacids (e. g., LA) are also molecules that can be easily transformed into important industrial intermediates and into a wide variety of fine chemicals. HHD is the product of HMF ring opening mediated by a hydrogenation, followed by an acid‐catalyzed ring opening in water. This tandem reaction can be promoted by bifunctional homogeneous catalysts soluble in water. They must be prone to catalyze the hydrogenation under H_2_ pressure and must be able to generate acid for the ring‐opening reaction. A series of iridium and ruthenium complexes were tested for this scope. The first example was developed by Zhang and co‐workers in 2015,^[104]^ where Cp*Ir complexes successfully catalyzed HMF ring opening in aqueous solution under mild conditions. A series of iridium complexes employing different ligands were synthetized and screened in the aqueous system. The best results obtained involved the catalyst **7**, which in the aqueous phase gained 85 % HHD yield with only 5 bar of H_2_ pressure (catalyst loading 0.26 mol %, 110 °C, 1 h; Scheme 15, conditions a).

A kinetic study showed that the consumption of HMF and HHD yield increased over time. BHMF concentration, however, remained constant until the end of the reaction, suggesting its role as an intermediate in HHD production.

The same group extended their study to the design and testing of iridium *ortho*‐hydroxyl and dimethylamino bipyridine‐functionalized complexes with the aim to enhance hydrogenation activity and water solubility of the catalyst.^[105]^ To test their influence on the catalytic performances, diversely substituted iridium *ortho*‐hydroxyl bipyridines (*o*‐OHbipy) were screened, all of them showing similar activity to **7** in the same operative conditions (catalyst loading 0.25 mol%, 10 bar H_2_, 120 °C). Nevertheless, lowering the catalyst loading to 0.0025 mol%, *o*‐OHbipy **8** displayed better performance than **7** at the same loading (Scheme 15, conditions b).

This behavior confirmed the expected hydroxy and dimethylamine influence. A deeper screening on aromatic ring substituents and reaction conditions stated that: (i) electron‐donating groups on the bipyridines enhanced catalytic activity; (ii) low pH (<2.5) has a detrimental effect on selectivity since aldol condensation of HMF into humins is favored in strongly acid conditions; and (iii) basic conditions favor hydrogenation but decrease HHD selectivity due to the low ring‐opening reaction rate. These further findings allowed to plot a three‐step reaction mechanism: hydrogenation of HMF to BHMF is followed by acid‐catalyzed ring opening to 1‐hydroxyhex‐3‐ene‐2,5‐dione (HHED), which undergoes consecutive hydrogenation.

Interestingly, employing 2 equiv. of formic acid as hydrogen source for the reaction, HMF was fully converted in 2 h by 0.005 mol % **9** and HHD was obtained with 61.4 % yield (TOF=6140 h^−1^) at 120 °C under N_2_ atmosphere (10 bar) (Scheme 15, conditions c).

**Scheme 16:**
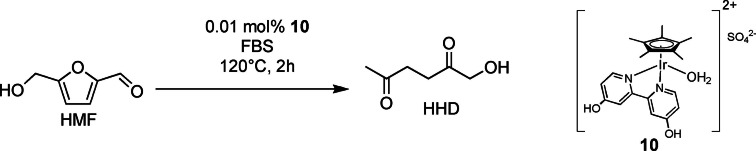
HMF reduction with a formic acid buffer solution mediated by iridium complex **10**.^[106]^

This mechanistic behavior was confirmed studying another class of similar iridium half‐sandwich bispyridines by Fu and co‐workers in 2016.^[106]^ Electronic and steric ligands effect and different reaction conditions were screened with the main aim to understand the catalyst behavior versus pH. Iridium complexes were thus tested in HMF reduction in formate and phosphate buffer solution (FBS, PBS) under mild reaction conditions. Analyzing HHD and BHMF yields (0.01 mol %  Ir, 120 °C, 2 h), the results showed that *para*‐situated electron‐donating substituents made the complexes more active in obtaining HHD. The best yield (92 %) in HHD was reached with catalyst **10** (loading 0.1 mol%, 130 °C, pH=2.5, 2 h) (Scheme 16).

The most interesting feature of this work is the information obtained on pH variation. Experiments demonstrated that at pH 4.5–7.0, reduction of the aldehyde groups was more favored than hydrolysis/ring‐opening reaction, hence leading to the generation of BHMF as the major product. At pH<4.0 (e. g., in PBS buffer at pH 2.5), BHMF could further react to open the furan ring or lead to the decomposition of BHMF. On the other hand, in FBS, the pH increases during time as formic acid is consumed as hydrogen source (from 2.5 to 6.5). Acidity in the initial steps of the reaction promotes the hydrolysis/ring‐opening reaction of BHMF, reducing its concentration and avoiding its polymerization. Then, hydrogenation is accelerated by the increase of pH, avoiding BHMF decomposition. These impressive results demonstrate that selectivity can be easily tuned, and that the reaction can be directed to the desired product exploiting formic acid as a double‐faced reactant: acid and the hydrogen source.

The authors also evaluated the possibility of recycling the catalyst thanks to a biphasic extraction at the end of the cycle. Even with a partial loss of catalyst during extraction, a 70 % HDD yield after the second cycle was obtained (92 % yield after the first cycle). The catalytic system was tested also on a crude HMF solution obtained from fructose: a 98 % yield of HDD (with respect to HMF) was reported. Due to the complexity of the reaction mechanism (ascribable to subsequent reactions that occur with different catalytic approaches, namely reduction and acidic hydrolysis) this is a field where homogeneous catalysis could compete and overcome heterogeneous methods even for real applications. Reaching elevated selectivity in HHD still deserves a fine tuning of ligands, metals, and acidic media to develop new and more active and selective systems.

**Scheme 17:**
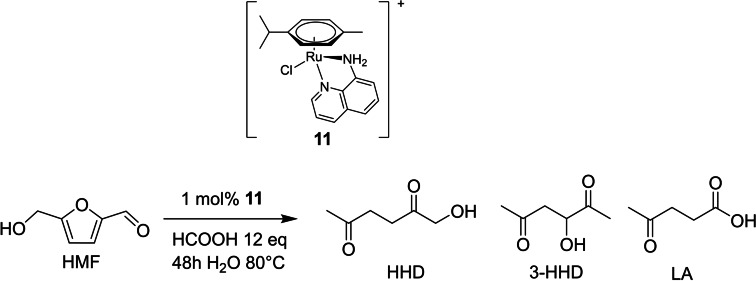
HMF reduction to hydroxydiketones and levulinic acid catalyzed by **11**.^[107]^

Singh and co‐workers in 2015 introduced the preparation of mixtures of HHD, 3‐HHD, and LA from HMF, catalyzed by arene‐ruthenium complexes bearing 8‐aminoquinoline in water, with formic acid as hydrogen source.^[107]^ Screening several complexes, the presence of a N−H moiety has been found to promote the formation of hydrogen bonds with the substrate, favoring proximity between the two and helping hydrogen shuttling. Thus, the catalyst **11** was the most active. After 48 h HMF was fully converted with 52 % 1‐HHD, 21 % LA, and 27 % 3‐HHD yield, respectively (80 °C, catalyst loading 1 mol%, formic acid 12 equiv.) (Scheme 17).

**Scheme 18:**
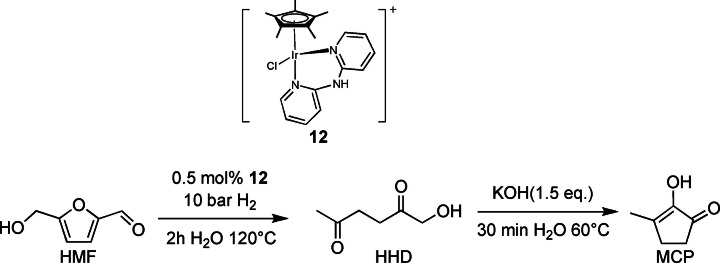
One‐pot synthesis of MCP from HMF mediated by iridium complex **12**.^[108]^

More recently, while searching for novel catalysts for the conversion of HMF to HHD, de Vries and co‐workers^[108]^ explored the activity of iridium and ruthenium catalysts. [Cp*Ir(dpa)Cl]Cl (dpa=dipyridylamine) (Scheme 18, complex **12**) was the most promising in this case; it showed not outstanding performance in comparison with the previously reported ones (loading 0.5 mol%, 10 bar H_2_, 120 °C, 69 % HHD yield). Nevertheless, these Ir catalysts were found active, for the first time in 2018, for the one‐pot transformation of HMF into the important building block 2‐hydroxy‐3‐methylcyclopent‐2‐enone (MCP). Fine tuning of operative conditions enabled an intramolecular condensation of HHD, which forms as intermediate, to MCP with 55 % yield (Scheme 18).

## 4 Production of Biobased HMF Derivatives by Reductive Amination

**Scheme 19:**
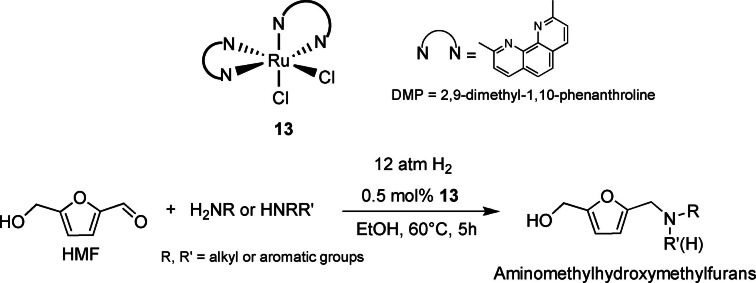
HMF amino reduction mediated by **13**.^[109]^

Zhang and co‐workers^[109]^ firstly applied homogeneous molecular catalysis in the direct reductive amination of HMF with amines promoted by Ru^II^ complexes. Among several tested complexes, dichlorobis(2,9‐dimethyl‐1,10‐phenanthroline) Ru^II^ [Ru(DMP)_2_Cl_2_] **13** (Scheme 19) resulted to be the most active catalyst. In general, bidentate strained ligands played an important role to control selectivity. The reaction was tested with H_2_ in a green solvent such as ethanol. The catalyst performed well on a broad substrate pool of primary, secondary, and aliphatic amines with fair to good yields (66–98 %). Optimized reaction conditions were stated studying the reactivity with aniline as following: catalyst loading 0.5 mol%, 60 °C 12 atm H_2_, 5 h, yield 98 %. Aminomethyl‐hydroxymethylfuran (AHF) derivatives are generally produced from furfuryl alcohol or furfural under harsh reaction conditions with low selectivity.

On the opposite, Cukalovic and Stevens reported a one‐pot, two‐step reductive amination of 5‐HMF in the absence of catalyst.^[110]^ However, this two‐step procedure starts with imine formation, which limits the scope of amine substrates, followed by the use of a NaBH_4_ excess that generates copious amounts of waste besides the costly hydrogenation reagent.

Direct hydrogenation of the imine derived from HMF and aniline was also studied, and 89 % of the corresponding AHF was obtained. This result supports the proposed mechanism, in which the direct reductive amination of HMF with amines proceeds via imine formation, followed by hydrogenation of the imine.

In a following work, the amination was carried out with an excess of HMF using primary aliphatic and benzyl amines at different temperature, H_2_ pressure, and catalyst loading.^[111]^ It led to the production of bis(hydroxylmethylfurfuryl)amine (BHMFA) as the major product. The reductive amination of HMF with primary amine was subsequently followed by reductive amination of HMF with the in‐situ generated secondary amine. BHMFA production has been optimized with *n*‐heptilamine in up to 90 % yield under the following reaction conditions: Ru(DMP)Cl_2_ **13** loading of 1 mol% at 110 °C with an excess of HMF (2.3 equiv.) under 20 bar of H_2_ for 12 h. Other Ru^II^ catalysts were tested with lower efficiency. Under optimized conditions, the substrate scope was investigated with various amines.

The nature of the amine also influenced the reaction output. For instance, the reactions of aliphatic straight‐chain amines such as *n*‐butyl and *n*‐dodecyl amine smoothly proceeded to generate the corresponding BHMFA in 79.6 and 82.6 % yield, respectively. On the contrary, employing ethyl and methyl amine as aqueous solutions (65 and 25 wt%), a serious detrimental effect was observed due to the presence of additional water that could suppress imine/iminium‐ion formation (yields 56.8 % and trace, respectively). Indeed, when methylamine hydrochloride was used the yield increased to 70.5 %. *Iso*‐butylamine gave the corresponding BHMFA in 75.1 % yield, whereas no product was observed using *tert*‐butylamine due to its steric hindrance. Finally, phenylethylamine led to 88.3 % yield in 1 h. The reactivity of benzyl amines confirmed the expected reactivity (product yields from 79.7 to 87.4 %) apart from *para*‐hydroxy functionalized phenyls that were found to be unreactive. Catalytic activity in this peculiar case was probably suppressed by coordination of the phenol group to the catalyst.

Scale‐up of the synthesis with *n*‐heptylamine was demonstrated up to gram quantity, interestingly maintaining an 84 % yield.

**Scheme 20:**
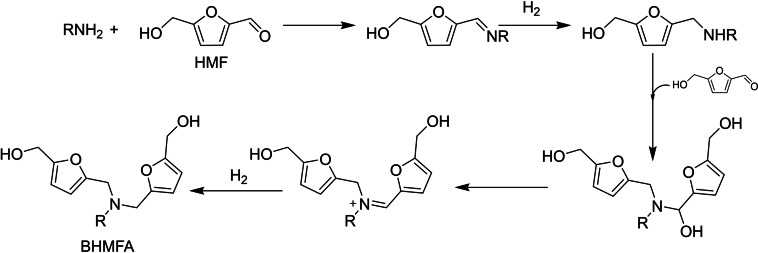
Proposed reaction pathway for the synthesis of BHMFA derivatives.^[111]^

To confirm the hypothesized reaction mechanism (Scheme 20), direct hydrogenation of imine was investigated. 95 % yield of secondary amine was obtained in this case. The latter reacted with HMF to produce tertiary amine BHMFA (90 % yield). The in‐situ generated secondary amine may further react with HMF to form an iminium ion intermediate, while the last step is the hydrogenation to form BHMFA.

These studies represent an important example of selective preparation of furan‐based monomers from bio‐based platform HMF under mild conditions. Having collected information for all the steps of the reaction, they pave the way toward the design of more efficient catalysts for economic production of BHMFAs.

**Scheme 21:**
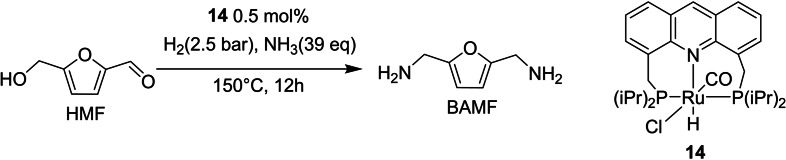
HMF amino reduction to BAMF catalyzed by **14**.^[114]^

Compared to polyesters, fewer new polyamides from renewables have been studied or introduced over the last decades.[^112^, ^113^] This is largely due to a lack of suitable routes to novel diamine monomers. Within this field Mecking and co‐workers^[114]^ converted HMF in 2,5‐bis(aminomethyl) furan (BAMF) in an elegant one‐pot direct amination/reductive amination using ammonia. The homogeneous catalyst [RuHCl(acridine‐*i*Pr‐PNP)(CO)] (Scheme 21, complex **14**), developed by Gunanathan and Milstein,^[115]^ was chosen within those available in literature active in “hydrogen borrowing” reactions that efficiently converts primary and secondary alcohols to primary amines.

The combined reaction requires the concurrent use of H_2_ and NH_3_ with a total pressure of 10 bar. Looking at the product distribution, the authors demonstrated the extremely fast consumption of HMF to the aminoalcohol (≈30 min) due to the easy conversion to imine and subsequent hydrogenation in the presence of excess of ammonia. Nevertheless, further conversion of the aminoalcohol to diamine is much slower. Due to saturation of the catalyst by H_2_, the catalyst itself is unavailable for dehydrogenation and the amount of aminoalcohol decreases very slowly (over 140 h), while the produced BAMF increases. Scaling up the process while keeping constant the hydrogen amount, the reaction was completed in 12 h.

However, BAMF is susceptible to several side reactions, especially at the high temperatures required for the amination process. For this reason, the selectivity of 85 % obtained at 150 °C (loading 0.5 mol%, HMF 10 mmol, NH_3_ 10 mL, 390 mmol, H_2_ 2.5) can be considered already satisfactory. Lowering the temperature to 140 °C solved the selectivity problem, but doubling the reaction time to 24 h was necessary.

This reaction is particularly attractive due to its elegance and complexity. However, catalysts able to work at low temperatures and pressures with short reaction time would be very desirable to decrease overall reaction cost.

Finally, a very recent work^[116]^ reports preliminary tests on the reactivity of HMF with aniline exploiting 2‐propanol as the hydrogen source. Reductive amination occurred with a 0.5 % loading of the catalyst (Ru‐MACHO‐BH), with MgSO_4_ (1 equiv.) at 90 °C giving a yield of 87 %.

## 5 Decarbonylation of HMF in the Presence of Compressed Carbon Dioxide

An integrated system exploited homogeneous Ir catalysts and the properties of compressed carbon dioxide in the selective decarbonylation of HMF to FA.^[117]^ Catalyst screening qualified the combination of [IrCl(cod)]_2_ (cod=1,5‐cyclooctadiene) (2.5 mol%) and tri‐*n*‐octylphosphine (PnOct_3_) (10 mol%), at 110 °C, 48 h in 1,4 dioxane as the best one yielding 48 % of FA with a selectivity of 98 %. To increase conversion and yield, the use of compressed CO_2_ was investigated with tris‐cyclohexyl (the best phosphine under the new conditions) pre‐coordinated to the iridium catalyst [Ir(CO)(PCy_3_)_2_Cl]. Quantitative conversion and 95 % selectivity in 12 h were obtained by adding 50 bar of CO_2_. The beneficial effect of CO_2_ on reactivity and selectivity was attributed to the formation of an expanded liquid phase and to the subsequent alteration of physicochemical properties of the solvent. Interestingly, the [Ir(CO)(PCy_3_)_2_Cl] complex can be recovered (86 %) and reused without a decrease in conversion and yield. In order to improve the applicability of the process, the selective conversion was then successfully scaled up to a HMF concentration of 12 wt% maintaining 98 % conversion with 88 % selectivity for FA. Furan‐derived, more sustainable solvents [such as 2‐methyltetrahydrofuran (2‐MTHF) and tetrahydrofurfuryl alcohol (THFA)] resulted in competitive conversions compared to 1,4‐dioxane but with a loss in FA selectivity (75 %). This result suggests that the use of compressed CO_2_ could also be exploited in other reactions of HMF.

## 6 Friedel‐Crafts Arylation of HMF

**Scheme 22:**
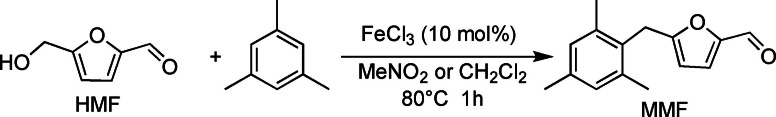
Iron‐catalyzed HMF arylation with mesitylene.^[118]^

After preliminary studies that briefly reported the FeCl_3_‐catalyzed solvent‐free Friedel‐Crafts reaction of HMF with *o*‐xylene, which provided a 37 % yield of the coupled product, the study by Zhou and Rauchfuss^[118]^ focused on reactions with mesitylene, which led to only one isomer. The product of arylation 5‐(mesitylmethyl)furfural (MMF), was obtained in high yields (up to 94 % in 1 h) when the reaction was conducted in MeNO_2_ or CH_2_Cl_2_ (FeCl_3_ loading 10 %, 80 °C) (Scheme 22).

The reaction is general as demonstrated with different arenes such as *p*‐xylene and toluene. Exploration of potentially greener solvents such as THF, *i*PrOH, *n*BuOH, and H_2_O was unsuccessful, and the useful solvents (MeNO_2_ and CH_2_Cl_2_) are unattractive solvents from a green perspective. Substitution of the Lewis acid catalyst with a Brønsted acid such as *p*‐toluenesulfonic acid (*p*‐TsOH) was tried to solve possible interaction between Fe^III^ and oxygenated solvents, resulting in the same detrimental effect in greener solvents reactivity like with FeCl_3_.

A final trial was the use of formic acid as a reactive solvent. HMF reached 94 % yield in MMF in 4 h at 120 °C. Addition of FeCl_3_ to the reaction mixture slightly accelerated the rate of reaction. However, the reaction was not enhanced to a rate that justified the use of further additives. One‐pot alkylation of mesitylene HMF represents a step forward diesel‐range liquids. However, it needs to be further developed in order to reach sustainable conditions.

Some other homogeneous HMF reactions catalyzed by bifunctional Lewis‐Brønsted acid catalysis in alcohols (metal chlorides) to furanyl ethers and alkyl levulinate esters have been extensively reviewed previously.^[119]^

## 7 Summary and Outlook

The production and utilization of 5‐hydroxymethylfurfural (HMF) can be deeply inserted into the concept of biorefinery, leading to the production of useful molecules that can find applications in different fields, starting from a green substrate generated from cellulose or sugars. HMF is a very versatile molecule due to the presence of different functionalities (hydroxide, aldehyde, and furan ring), that can all be converted into other moieties. Thus, HMF has been subjected to selective oxidations, hydrogenations, reductive aminations, etherifications, acetalizations, and decarbonylation reactions, among others. This leads to the production of a wide pool of molecules with different applications as fuels, monomer, pharmaceutical precursors, and so on. Although heterogeneous catalysis is the usual choice for these processes, homogeneous catalysis can provide different advantages and has the potential to solve some of the issues found in classical processes. In this Review, the development and application of homogeneous catalysts and catalytic systems to HMF conversion has been critically reviewed. Homogeneous catalysis has been applied to different HMF transformations (selective oxidations, hydrogenations, reductive aminations, etherifications, acetalizations, and decarbonylation) with interesting results in terms of milder reaction conditions and easily tunable selectivity have been discussed. This highlights homogeneous catalysis as a good prospect to boost the biorefinery development, especially where high selectivities are compulsory. However, to fully exploit its potential, some important fields must be further or newly investigated. The state of the art is generally underdeveloped but gives important parameters and critical directions toward the use of greener conditions (e. g., sustainable solvents, lower temperature, lower catalyst loadings) and, above all, suitability for efficient recycling, the principal Achilles heel for homogeneous catalysis in industrial application. Furthermore, most of the employed molecular catalysts are based on noble and expensive metals, whose utilization must be reduced in a circular economy perspective, moving toward the development of new Earth‐abundant metal molecular catalysts. Interestingly, some HMF‐based processes already employed for the synthesis of bio‐based platform in heterogeneous catalysis are still unexplored from the homogeneous side, leaving room for the development of novel approaches. Stability, another critical point in industrial catalytic processes, is scarcely appointed in the state of the art. Finding new solutions to solve this problem will also favor recovery and recyclability. With this aim in mind, immobilization of catalysts over insoluble supports or the use of biphasic conditions could be suitable routes that would lead to the merging of the advantages of homogeneous and heterogeneous catalysis toward stable and easily recoverable molecular catalysts.

## Conflict of interest

The authors declare no conflict of interest.

## Biographical Information

*Rita Mazzoni received her Ph.D in Chemistry in 2005 from the University of Bologna working on the design of rhodium homogeneous catalysts applied in hydroformylation and hydrosilylation. Currently she holds a position as Associate Professor in Inorganic Chemistry at the Department of Industrial Chemistry (University of Bologna). Her main research interests are focused on the rational design of homogeneous catalytic systems for sustainable biomass conversion (e. g., bio‐oil mixtures, HMF and bio‐ethanol valorization) and energy transition (water oxidation, hydrogen production, aqueous‐phase reforming) based on noble and Earth‐abundant transition metal complexes that combine cyclopentadienone and N‐heterocyclic carbene ligands*.

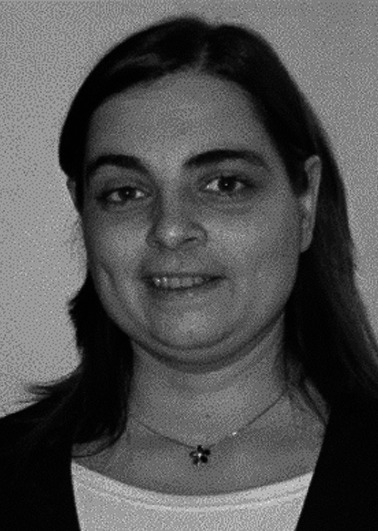

## Biographical Information

*Alessandro Messori obtained his Master's degree in Industrial chemistry at the University of Bologna in 2020. After graduation he started his Ph.D. under the supervision of Prof. Mazzoni in inorganic chemistry, employing Earth‐abundant metallic complexes as catalysts for sustainable reactions. He is currently studying manganese, iron, rhenium, and ruthenium N‐heterocyclic carbene complexes in alcohols homologation, water oxidation, and aqueous‐phase reforming of glucose. Another important part in his research is devoted to the heterogenization of homogeneous catalytic systems to ease recovery and reuse of the catalyst*.

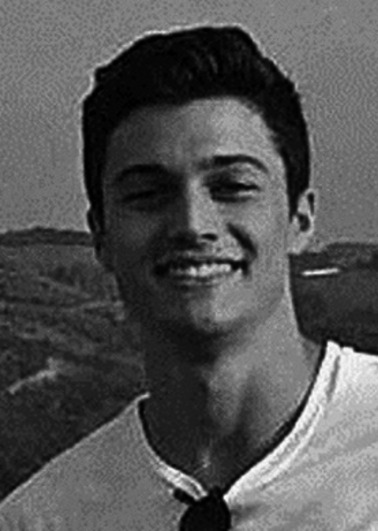

## Biographical Information

*Andrea Fasolini obtained is Master's degree in Industrial Chemistry from the University of Bologna in 2016. He then started his Ph.D. in the laboratory of catalytic processes development with a project on the production of pure hydrogen from methane using membrane reactors. He also took part in projects to produce green hydrogen in liquid and gas phase. He obtained his Ph.D. in 2020 and continued his career as a Postdoc fellow starting new projects regarding CO_2_ utilization, electrocatalysis, photocatalysis, and photoelectrocatalysis. In 2022 he became Research Fellow of the catalytic process development of the University of Bologna*.